# Extracellular Vesicles from Buccal Fat Pad-Derived Dedifferentiated Fat Cells Modulate Inflammatory Macrophage Responses and Attenuate Temporomandibular Joint Osteoarthritis

**DOI:** 10.3390/ijms27209389

**Published:** 2026-10-21

**Authors:** Hayato Oku, Yusuke Nishiguchi, Tomofumi Yamamoto, Jun-Ichiro Jo, Yoshiya Hashimoto, Hirohito Kubo, Toshihiko Takenobu

**Affiliations:** 1 Second Department of Oral and Maxillofacial Surgery, Osaka Dental University, 8-1 Kuzuhahanazonocho, Hirakata 573-1121, Osaka, Japan; oku-h@cc.osaka-dent.ac.jp (H.O.); takenobu-t@cc.osaka-dent.ac.jp (T.T.); 2 Division of Biological Chemistry and Biologicals, National Institute of Health Sciences, 3-25-26 Tonomachi, Kawasaki-ku, Kawasaki 210-9501, Kanagawa, Japan; toyamamo@nihs.go.jp; 3 Department of Biomaterials, Osaka Dental University, 8-1 Kuzuhahanazonocho, Hirakata 573-1121, Osaka, Japan; jo-j@cc.osaka-dent.ac.jp (J.-I.J.); yoshiya@cc.osaka-dent.ac.jp (Y.H.); 4 First Department of Oral and Maxillofacial Surgery, Osaka Dental University, 8-1 Kuzuhahanazonocho, Hirakata 573-1121, Osaka, Japan; kubo-h@cc.osaka-dent.ac.jp

**Keywords:** temporomandibular joint osteoarthritis, dedifferentiated fat cells, extracellular vesicles, macrophages, inflammation, intra-articular injection

## Abstract

Temporomandibular joint osteoarthritis (TMJ-OA) involves inflammation and degeneration of mandibular condylar cartilage and subchondral bone. Dedifferentiated fat cells (DFATs) were established from buccal fat pad tissue obtained from a single human donor. We examined the effects of DFAT-derived extracellular vesicle preparations (DFAT-EVs) on inflammatory macrophage responses in vitro and disease-related outcomes in TMJ-OA in vivo. DFAT-EVs were applied to lipopolysaccharide- and interferon-γ-stimulated RAW264.7 macrophages and administered intra-articularly in rats with monosodium iodoacetate-induced TMJ-OA. Fluorescence signals from labeled DFAT-EVs were observed in RAW264.7 cells. DFAT-EVs reduced nitric oxide production and modulated nitric oxide synthase 2, interleukin (IL)-1β, IL-6, IL-10, IL-4, and tumor necrosis factor-α responses, although effects varied among inflammatory mediators. Head withdrawal threshold was significantly higher in the DFAT-EVs group than in the OA group at several time points in the 6-week cohort, but not in the 4-week cohort. Administration of DFAT-EVs attenuated the reduction in mandibular condylar bone volume fraction and was associated with preservation of cartilage architecture and matrix. Osteoarthritis Research Society International scores were significantly lower in the DFAT-EVs group than in the OA group at 4 weeks but not at 6 weeks. These in vitro and in vivo findings support further evaluation of DFAT-EVs for TMJ-OA.

## 1. Introduction

Temporomandibular joint osteoarthritis (TMJ-OA) affects the entire joint and is accompanied by deterioration of condylar cartilage, disturbed turnover of subchondral bone, and inflammation of the synovium [1,2,3,4]. Clinically, TMJ-OA causes joint pain and impaired mandibular function, adversely affecting patients’ quality of life [1,2,5]. Synovial inflammatory responses, including macrophage activation and inflammatory signaling in synovial fibroblasts, disrupt joint-tissue homeostasis [2,3,6,7,8,9]. Among the mediators associated with this process are interleukin (IL)-1β, IL-6, and nitric oxide (NO), the latter generated by inducible nitric oxide synthase (iNOS; encoded by nitric oxide synthase 2 [NOS2]) [2,3,6,8,10,11,12]. Accordingly, therapeutic strategies that control intra-articular inflammation while protecting cartilage and subchondral bone from progressive structural damage are needed [1,2,4].

Conservative treatments, including pharmacological therapy, occlusal splints, and physical therapy, are generally used as first-line management for TMJ-OA [2,5,13,14]. For persistent symptoms, arthrocentesis, with or without intra-articular hyaluronic acid or corticosteroid injection, may reduce pain and improve mandibular function; however, the additional benefit of injection remains uncertain [13,14]. Evidence that these minimally invasive treatments achieve sustained restoration of degenerated cartilage or subchondral bone is limited [1,14]. Thus, currently available treatments are primarily symptomatic, and no intra-articular therapy reliably controlling inflammation and preventing progressive structural damage in TMJ-OA has been clinically established [1,13].

Mesenchymal stem/stromal cells (MSCs) have been explored for their potential to combine immunomodulation with joint repair. Although intra-articular MSC administration has demonstrated anti-inflammatory and chondroprotective effects in preclinical TMJ-OA models, its effects on subchondral bone have varied among models and treatment protocols [1,15]. Moreover, clinical translation is complicated by variability in MSC properties and potency based on the tissue source, donor, culture conditions, and passage number [16]. Furthermore, controlling the survival, retention, and behavior of administered cells is challenging, and viable-cell products pose challenges related to storage, transportation, manufacturing, quality control, and safety [16,17,18]. Growing evidence suggests that the therapeutic effects of MSCs are mediated largely by paracrine signaling through soluble factors and extracellular vesicles (EVs), rather than by engraftment and differentiation alone [16,17,18,19,20,21,22,23,24,25].

MSC-derived EVs, which are membrane-enclosed particles that carry bioactive molecules and mediate intercellular communication, are being investigated as cell-free mediators of MSC paracrine activity [16,18,19,20,21,22,23,24,25,26]. Although intra-articular administration of MSC-derived EVs attenuated inflammatory and catabolic responses as well as cartilage and subchondral bone degeneration in a rat model of TMJ-OA [27], EV composition and biological activity depend on the origin, phenotype, and culture state of the parental cells [16,26,28,29]. Therefore, careful selection of the parental cell source and control of culture conditions are essential for developing reproducible EV-based therapeutics [16,26,28].

Dedifferentiated fat cells (DFATs), generated from mature adipocytes by ceiling culture, form a relatively homogeneous cell population with sustained proliferative capacity and multilineage differentiation potential [30,31,32,33]. Buccal fat is readily accessible and can provide these cells with limited morbidity at the harvest site [31,33,34]. In our previous study, EVs derived from human buccal fat pad-derived DFATs (DFAT-EVs) were characterized by their morphology, size distribution, and expression of the EV surface markers CD9 and CD63 [34]. CD81 expression in DFAT-EVs has also been reported [35]. However, their effects on inflammatory macrophage responses, mechanical hypersensitivity, cartilage degeneration, and subchondral bone alterations in TMJ-OA remain unclear.

In the present study, we investigated how DFAT-EVs modulate inflammatory macrophage responses and attenuate mechanical hypersensitivity and structural joint damage in TMJ-OA. Inflammation-related responses were examined in RAW264.7 macrophages stimulated with lipopolysaccharide (LPS) and interferon (IFN)-γ. Effects of intra-articular DFAT-EV administration were assessed using the head withdrawal threshold (HWT), mandibular condylar cartilage degeneration, and subchondral bone microarchitecture in a monosodium iodoacetate (MIA)-induced rat model of TMJ-OA [36].

## 2. Results

### 2.1. Establishment, Morphology, and Surface Marker Expression of DFATs

**Figure 1:**
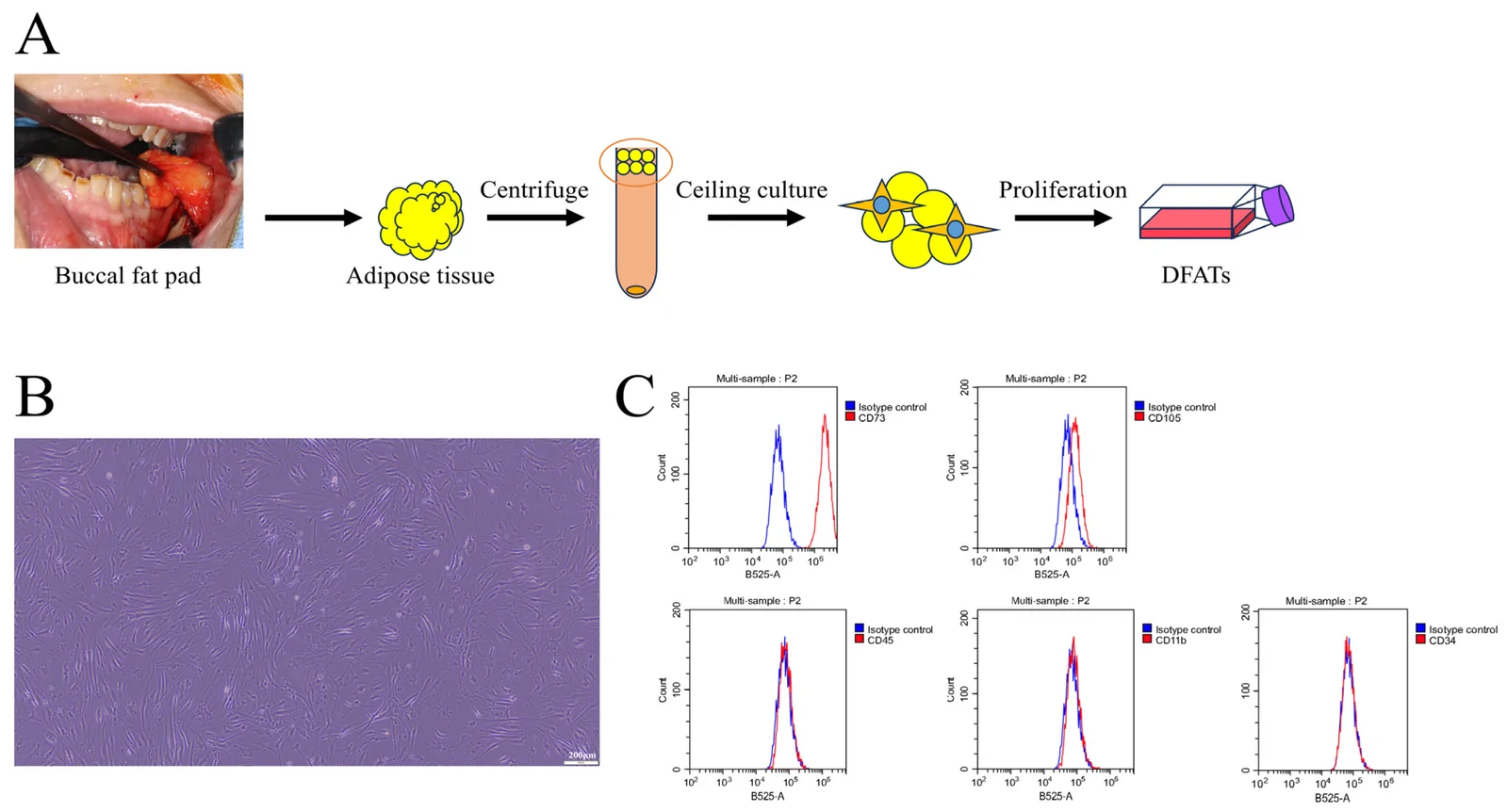
Generation and phenotypic characterization of dedifferentiated fat cells (DFATs). (**A**) Workflow for deriving DFATs from human buccal adipose tissue. Buccal fat pad samples obtained at Osaka Dental University Hospital underwent enzymatic dissociation in 0.1% collagenase type I. Centrifugal separation was then used to recover the floating mature adipocytes. These cells were cultured by the ceiling culture technique in flasks completely filled with Dulbecco’s modified Eagle medium supplemented with 20% fetal bovine serum. The flasks were subsequently inverted, and conventional culture was continued to establish DFATs. (**B**) Representative image of human DFATs after ceiling culture. The cells exhibited a fibroblast-like morphology. Scale bar: 200 μm. (**C**) Flow cytometric analysis of DFATs. DFATs were positive for CD73 and CD105 and negative for CD11b, CD34, and CD45.

Ceiling culture of mature adipocytes obtained from the human buccal fat pad yielded proliferating DFATs (Figure 1A). During subsequent culture, the cells attached to the culture surface and displayed a spindle-shaped, fibroblast-like appearance (Figure 1B). Surface-antigen profiling by flow cytometry showed expression of CD73 and CD105, whereas CD11b, CD34, and CD45 were not detected (Figure 1C).

### 2.2. Characterization of DFAT-EVs and Fluorescence Associated with the Labeled DFAT-EV Preparation

**Figure 2:**
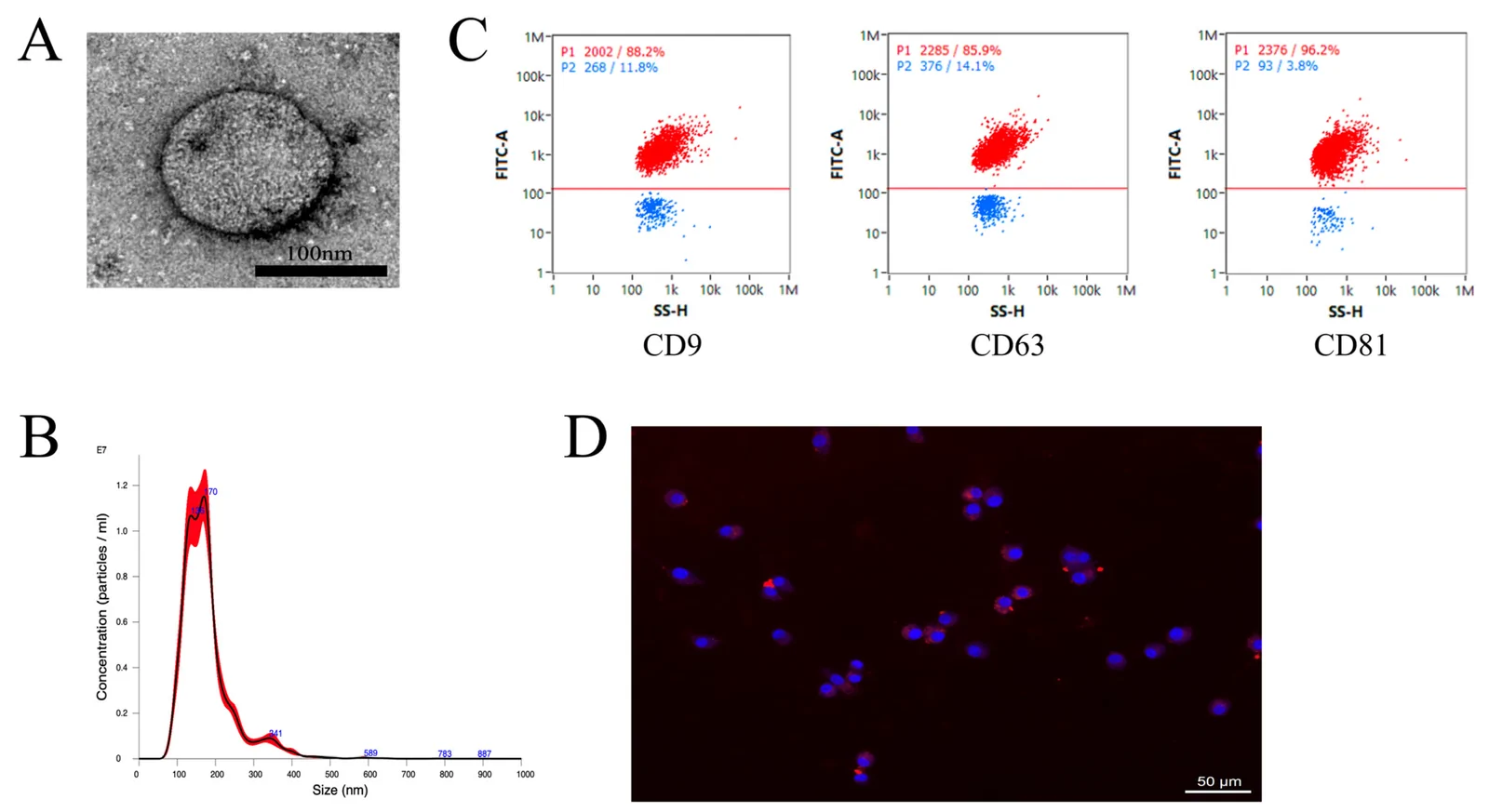
Fluorescence associated with the labeled DFAT-EV preparation. (**A**) Representative transmission electron microscopy image of DFAT-EVs. Scale bar: 100 nm. (**B**) Particle size distribution and particle concentration of DFAT-EVs determined by nanoparticle tracking analysis. (**C**) Surface marker expression of DFAT-EVs determined by nano-flow cytometric analysis. DFAT-EVs were positive for CD9, CD63, and CD81. The red horizontal lines indicate the fluorescence thresholds used to distinguish marker-positive events (P1, red) from marker-negative events (P2, blue). (**D**) Fluorescence microscopy images of RAW264.7 macrophages incubated with the labeled DFAT-EV preparation. Scale bar: 50 μm.

Transmission electron microscopy showed particles with a spherical, membrane-bound vesicular morphology (Figure 2A). Nanoparticle tracking analysis yielded a mean particle diameter of 177.0 ± 0.6 nm and particle concentration of 1.27 × 10^9^ particles/mL (Figure 2B). Nano-flow cytometry further demonstrated the presence of the EV-associated surface markers CD9, CD63, and CD81 (Figure 2C). Overall, the morphological, particle-size, and surface-marker findings corresponded to characteristics previously described for EVs.

Fluorescence microscopy showed intracellular red fluorescence signals in RAW264.7 macrophages after incubation with the labeled DFAT-EV preparation (Figure 2D). Because a dye-only control was not included, these findings were interpreted as the detection of fluorescence from the labeled preparation rather than as definitive evidence of EV-specific cellular uptake.

### 2.3. Effects of DFAT-EVs on Inflammatory Responses in RAW264.7 Macrophages

**Figure 3:**
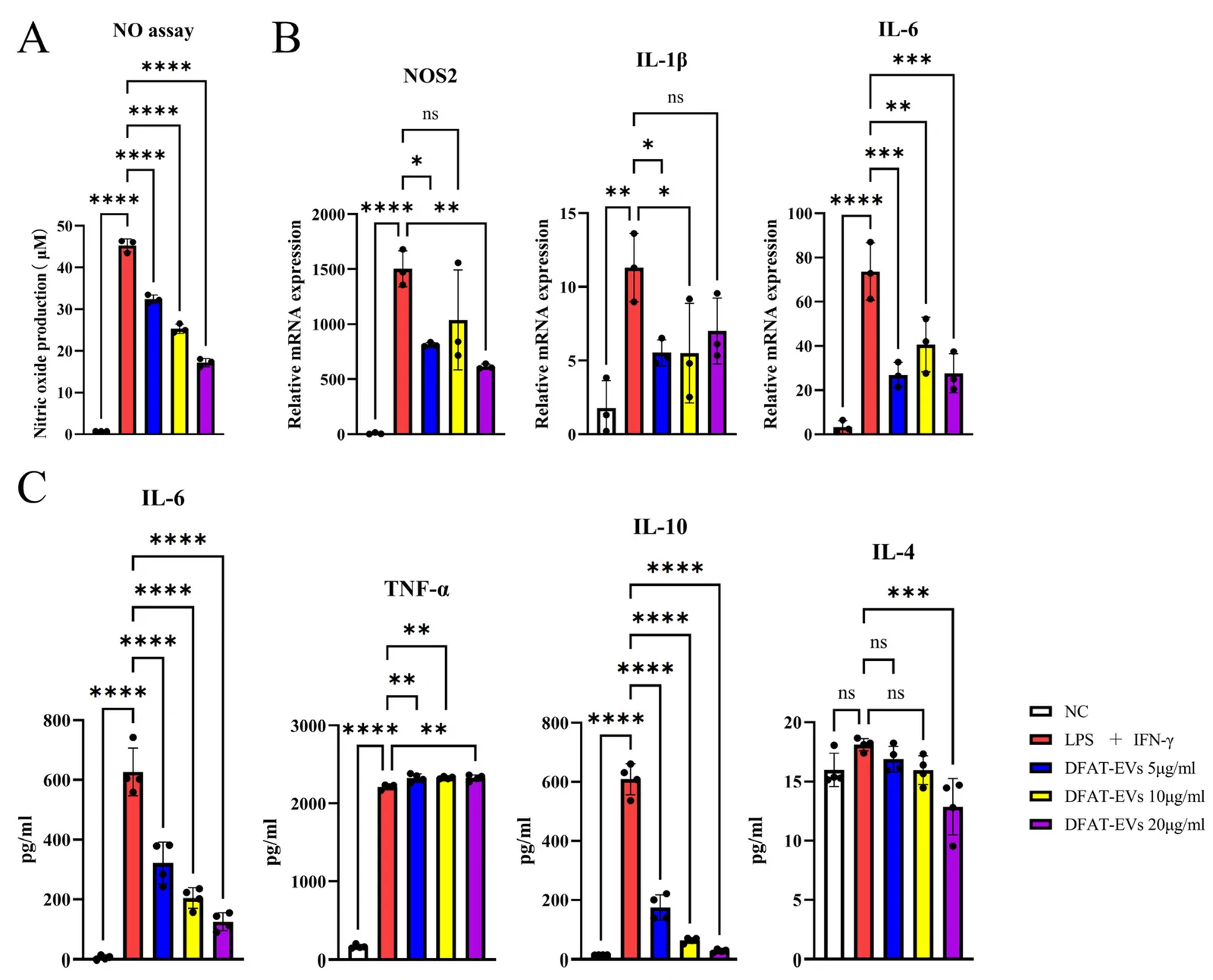
DFAT-EVs modulated inflammation-related responses in lipopolysaccharide (LPS)- and interferon (IFN)-γ-stimulated RAW264.7 macrophages. RAW264.7 macrophages were stimulated with LPS (100 ng/mL) and IFN-γ (20 ng/mL) and treated with DFAT-EVs (5, 10, or 20 μg/mL). (**A**) NO production in the culture supernatants was measured using the Griess assay. (**B**) NOS2, IL-1β, and IL-6 mRNA expression levels were analyzed using quantitative real-time PCR. (**C**) IL-6, tumor necrosis factor (TNF)-α, IL-10, and IL-4 concentrations in the culture supernatants were measured using enzyme-linked immunosorbent assay. Data are presented as the mean ± standard deviation [(**A**,**B**), *n* = 3 independent experiments; (**C**), *n* = 4 independent experiments]. Independent experiments were performed using separately prepared EV batches derived from DFATs obtained from the same human donor. * *p* < 0.05, ** *p* < 0.01, *** *p* < 0.001, and **** *p* < 0.0001; ns, not significant.

NO production in culture supernatants was evaluated using the Griess assay. Stimulation with LPS and IFN-γ significantly increased NO production in RAW264.7 macrophages compared with the negative control (NC) group (*p* < 0.0001). DFAT-EVs treatment significantly reduced NO production at all three tested concentrations (*p* < 0.0001 for each), with the lowest level observed at 20 μg/mL (Figure 3A).

Stimulation with LPS and IFN-γ significantly increased NOS2, IL-1β, and IL-6 mRNA expression in RAW264.7 macrophages compared with that in the NC group (*p* < 0.0001, *p* = 0.0024, and *p* < 0.0001, respectively). NOS2 mRNA expression was significantly reduced to approximately 54% and 41% of that in the LPS + IFN-γ group following treatment with DFAT-EVs at 5 and 20 μg/mL, respectively (*p* = 0.0117 and *p* = 0.0020, respectively). Although NOS2 mRNA expression was reduced to approximately 69% of that in the LPS + IFN-γ group following treatment with DFAT-EVs at 10 μg/mL, the difference was not statistically significant (*p* = 0.0962). Treatment with DFAT-EVs at 5 and 10 μg/mL significantly reduced IL-1β mRNA expression to approximately 49% of that observed in the LPS + IFN-γ group (*p* = 0.0335 and *p* = 0.0305, respectively). Although IL-1β mRNA expression was reduced to approximately 63% of that in the LPS + IFN-γ group following treatment with DFAT-EVs at 20 μg/mL, the difference was not statistically significant (*p* = 0.1606). Treatment with DFAT-EVs at 5, 10, and 20 μg/mL significantly reduced IL-6 mRNA expression to approximately 37–55% of that observed in the LPS + IFN-γ group (*p* = 0.0004, *p* = 0.0060, and *p* = 0.0005, respectively; Figure 3B).

Stimulation with LPS and IFN-γ significantly increased IL-6, tumor necrosis factor (TNF)-α, and IL-10 concentrations in the culture supernatants compared with those in the NC group (*p* < 0.0001 for all cytokines). DFAT-EV treatment at 5, 10, and 20 μg/mL significantly reduced IL-6 and IL-10 concentrations compared with the LPS + IFN-γ group (*p* < 0.0001 for all concentrations), with the lowest concentrations being at 20 μg/mL. In contrast, TNF-α concentrations increased slightly but significantly after DFAT-EV treatment at 5, 10, and 20 μg/mL (*p* = 0.0018, *p* = 0.0014, and *p* = 0.0017, respectively). LPS and IFN-γ stimulation did not significantly alter IL-4 concentrations relative to the NC group (*p* = 0.1990). DFAT-EV treatment at 5 and 10 μg/mL did not significantly alter IL-4 concentrations relative to the LPS + IFN-γ group (*p* = 0.6900 and *p* = 0.1952, respectively), whereas treatment at 20 μg/mL significantly reduced the IL-4 concentration (*p* = 0.0005; Figure 3C).

### 2.4. HWT

**Figure 4:**
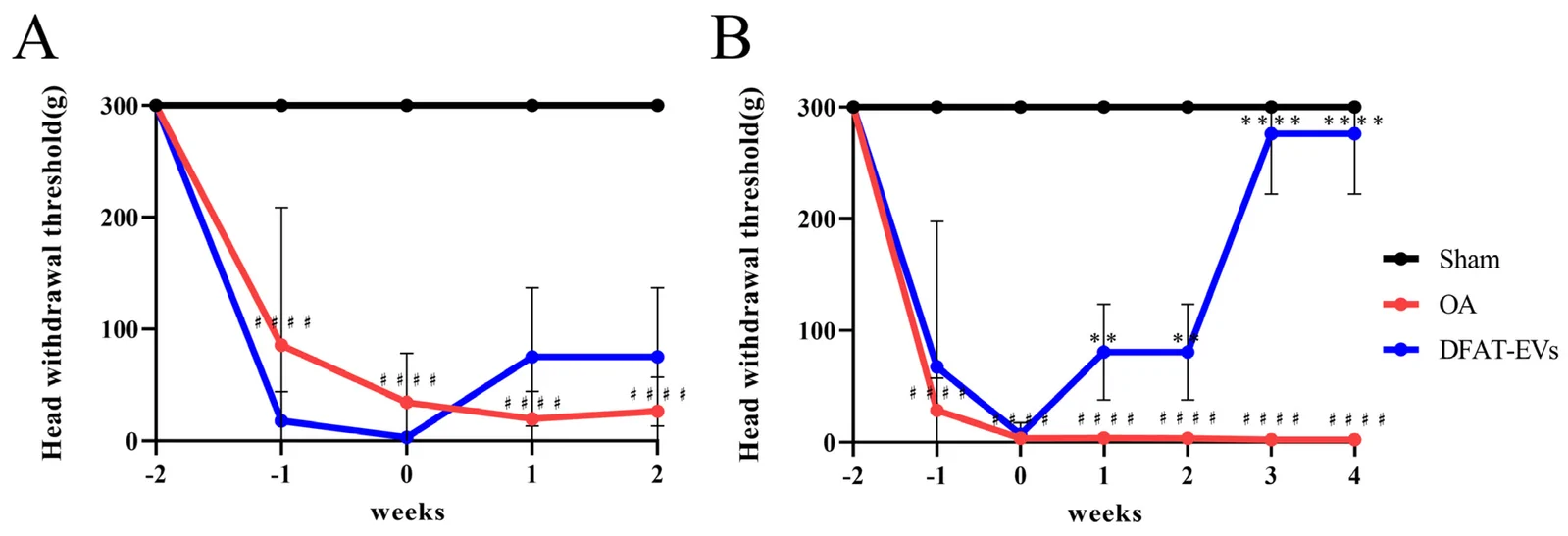
Longitudinal analysis of mechanical hypersensitivity following administration of DFAT-EVs. (**A**) Longitudinal changes in the head withdrawal threshold (HWT) in the 4-week evaluation cohort. The HWT was measured once weekly beginning on the day of monosodium iodoacetate (MIA) administration. On the horizontal axis, week 0 represents the time of the first DFAT-EVs or phosphate-buffered saline (PBS) administration, which was performed 2 weeks after MIA administration. The animals were evaluated until 4 weeks after MIA administration. Data are presented as the mean ± standard deviation (SD). #### *p* < 0.0001 vs. the Sham group; *n* = 5 per group. (**B**) Longitudinal changes in the HWT in the 6-week evaluation cohort. The HWT was measured once weekly beginning on the day of MIA administration. On the horizontal axis, week 0 represents the time of the first DFAT-EVs or PBS administration, which was performed 2 weeks after MIA administration. The animals were evaluated 6 weeks after MIA administration. Data are presented as the mean ± SD. #### *p* < 0.0001 vs. the Sham group; ** *p* < 0.01 and **** *p* < 0.0001 vs. the OA group. *n* = 5 per group.

HWT was measured at each time point after MIA administration to evaluate mechanical hypersensitivity. In the 4-week evaluation cohort, HWTs in the OA and DFAT-EVs groups were significantly lower than those in the Sham group after MIA administration, indicating mechanical hypersensitivity (*p* < 0.0001). In the OA group, the reduced HWT persisted throughout the evaluation period. HWT did not differ significantly between the DFAT-EV and OA groups at 2, 3, or 4 weeks after MIA administration (*p* = 0.4792, *p* = 0.1055, and *p* = 0.1740, respectively; Figure 4A). In the 6-week evaluation cohort, HWTs in the OA and DFAT-EV groups were also significantly lower than those in the Sham group after MIA administration (*p* < 0.0001). In the OA group, the reduced HWT persisted throughout the evaluation period. HWT was significantly higher in the DFAT-EV group than in the OA group at 3 and 4 weeks (*p* = 0.0033 and *p* = 0.0031, respectively) and remained higher at 5 and 6 weeks after MIA administration (*p* < 0.0001 for both time points; Figure 4B).

### 2.5. Micro-Computed Tomography Analysis

**Figure 5:**
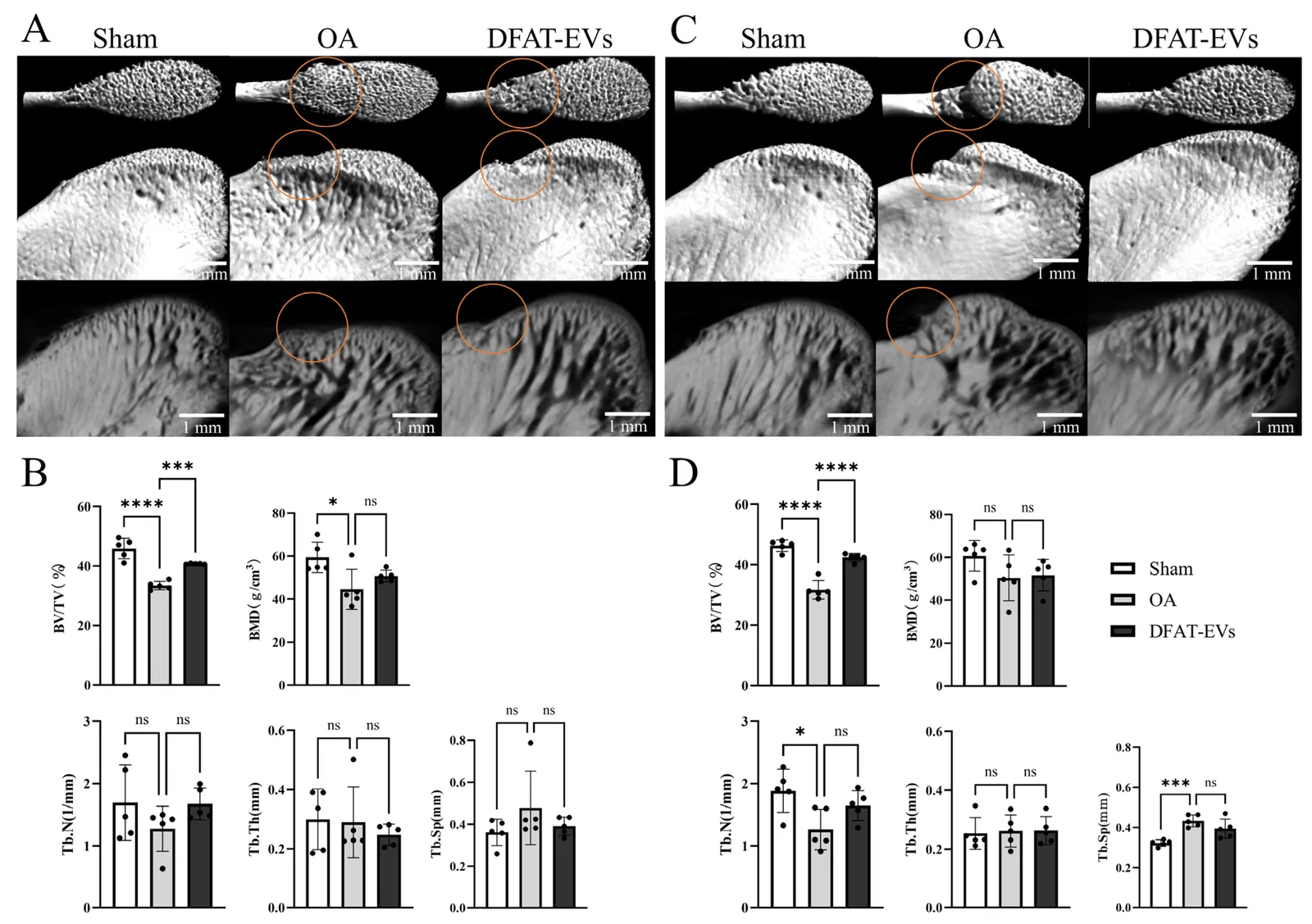
Micro-computed tomography (micro-CT) analysis of the mandibular condyle at 4 and 6 weeks after monosodium iodoacetate (MIA) administration. Representative three-dimensional (3D) reconstructions, enlarged 3D reconstructions, and sagittal micro-CT images of the mandibular condyles in the Sham, OA, and DFAT-EV groups are shown. In each image panel, the upper row shows 3D reconstructions, the middle row shows enlarged 3D reconstructions of the condylar surface, and the lower row shows sagittal micro-CT images. (**A**) Representative images at 4 weeks after MIA administration. Orange circles indicate areas of deformity, including irregularities of the condylar surface and alterations in bone morphology. Scale bar: 1 mm. (**B**) Quantitative micro-CT analysis at 4 weeks after MIA administration. Bone volume fraction (BV/TV), bone mineral density (BMD), trabecular number (Tb.N), trabecular thickness (Tb.Th), and trabecular separation (Tb.Sp) were analyzed. Data are presented as the mean ± standard deviation (SD). * *p* < 0.05 and **** *p* < 0.0001 vs. the Sham group; *** *p* < 0.001 vs. the OA group; ns, not significant. *n* = 5 per group. (**C**) Representative images at 6 weeks after MIA administration. Orange circles indicate areas of deformity, including irregularities of the condylar surface and alterations in bone morphology. Scale bar: 1 mm. (**D**) Quantitative micro-CT analysis at 6 weeks after MIA administration. BV/TV, BMD, Tb.N, Tb.Th, and Tb.Sp were analyzed. Data are presented as the mean ± SD. * *p* < 0.05, *** *p* < 0.001, and **** *p* < 0.0001 vs. the Sham group; **** *p* < 0.0001 vs. the OA group; ns, not significant. *n* = 5 per group.

Micro-Computed Tomography (Micro-CT) demonstrated a reduction in bone volume fraction (BV/TV) in the OA group relative to the Sham group at 4 and 6 weeks after MIA administration (*p* < 0.0001 at both time points). DFAT-EV administration partly mitigated this reduction, as BV/TV was higher in the DFAT-EV group than in the OA group at 4 weeks (*p* = 0.0003) and 6 weeks (*p* < 0.0001; Figure 5B,D). At 4 weeks, bone mineral density (BMD) was lower in the OA group than in the Sham group (*p* = 0.0109). BMD was numerically higher in the DFAT-EV group than in the OA group, but this difference was not significant (*p* = 0.3384; Figure 5B). No significant differences in BMD were detected at 6 weeks (Figure 5D). None of the trabecular parameters, including trabecular number (Tb.N), trabecular thickness (Tb.Th), and trabecular separation (Tb.Sp), differed significantly among the groups at 4 weeks. At 6 weeks, Tb.N was lower (*p* = 0.0154) and Tb.Sp was higher (*p* = 0.0005) in the OA group than in the Sham group. Compared with the OA group, the DFAT-EV group showed numerically higher Tb.N and lower Tb.Sp values; however, these differences were not statistically significant (*p* = 0.1362 and *p* = 0.1760, respectively). Tb.Th did not differ significantly among the groups at either time point (Figure 5B,D).

### 2.6. Histological Evaluation

**Figure 6:**
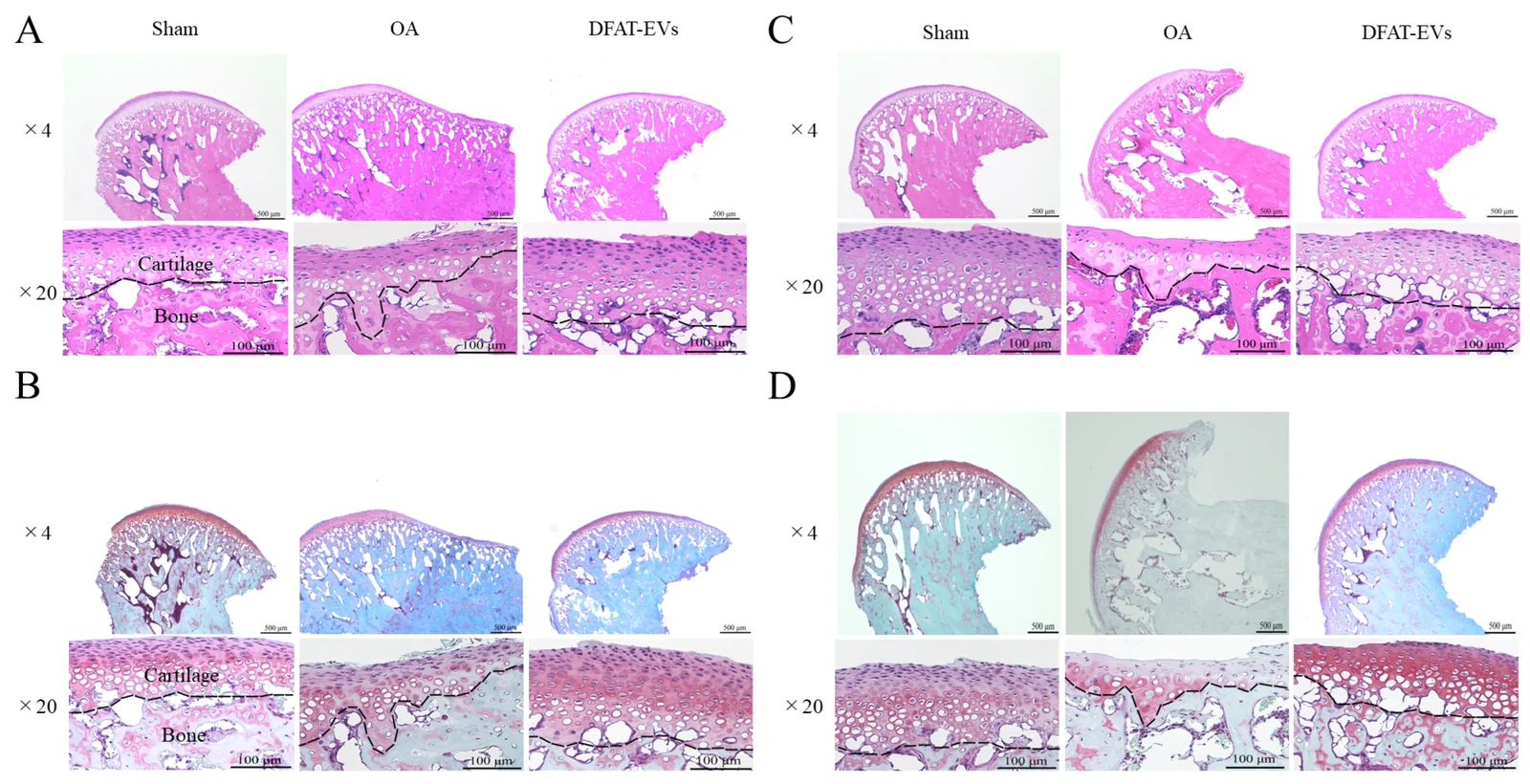
Histological findings in the mandibular condyle 4 and 6 weeks after monosodium iodoacetate administration. Representative sections of the mandibular condyles in the Sham, OA, and DFAT-EV groups stained with hematoxylin and eosin or Safranin O/Fast Green are shown. Panels (**A**,**B**) correspond to the 4-week endpoint, whereas panels (**C**,**D**) correspond to the 6-week endpoint. Black dashed lines delineate the interface between the mandibular condylar cartilage and underlying subchondral bone. Images in the upper and lower rows were acquired at ×4 and ×20 magnification, respectively. Scale bars represent 500 μm in the upper-row images and 100 μm in the lower-row images.

At 4 and 6 weeks after MIA administration, hematoxylin and eosin staining showed a smooth articular surface, preserved cartilage architecture, and organized chondrocytes in the Sham group. In contrast, the OA group exhibited an irregular mandibular condylar cartilage surface, disrupted cartilage layer, chondrocyte disorganization, and structural alterations in subchondral bone. These histological changes were less pronounced in the DFAT-EV group, with relative preservation of cartilage architecture. Safranin O staining was reduced in the OA group but relatively preserved in the DFAT-EV group at both time points, consistent with relative preservation of the cartilage matrix (Figure 6A–D).

### 2.7. Osteoarthritis Research Society International Scores

**Figure 7:**
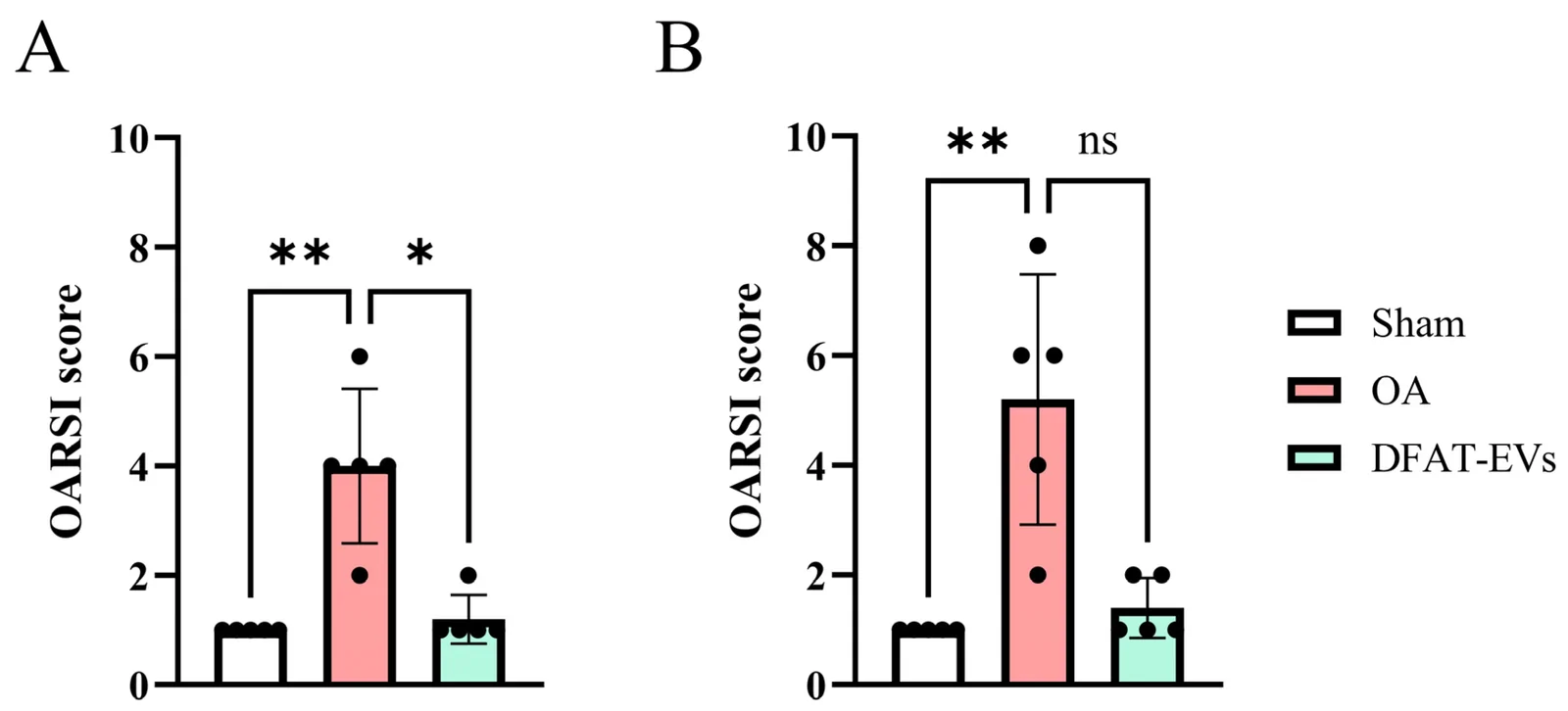
Osteoarthritis Research Society International (OARSI) scores in the mandibular condylar cartilage. (**A**) OARSI scores in the Sham, OA, and DFAT-EV groups at 4 weeks after MIA administration. The score was significantly higher in the OA group than in the Sham group, and was significantly lower in the DFAT-EV group than in the OA group. (**B**) OARSI scores in the Sham, OA, and DFAT-EV groups at 6 weeks after MIA administration. The score was significantly higher in the OA group than in the Sham group, whereas no significant difference was detected between the OA and DFAT-EV groups. Data are presented as individual values with the median and interquartile range. Statistical analysis was performed using the Kruskal–Wallis test followed by Dunn’s multiple-comparisons test. * *p* < 0.05 and ** *p* < 0.01; ns, not significant. *n* = 5 per group.

Quantitative histological scoring revealed significant overall differences among the groups at both evaluation points (Kruskal–Wallis test: *p* = 0.0020 at 4 weeks and *p* = 0.0041 at 6 weeks). All Sham animals had an Osteoarthritis Research Society International (OARSI) score of 1 because minor surface irregularities were classified as grade 1 and stage 1. At 4 weeks, the OARSI score was significantly higher in the OA group than in the Sham group (Dunn’s adjusted *p* = 0.0046) and was significantly lower in the DFAT-EV group than in the OA group (adjusted *p* = 0.0191; Figure 7A). At 6 weeks, the OARSI score was significantly higher in the OA group than in the Sham group (adjusted *p* = 0.0041), whereas the difference between the DFAT-EV and OA groups did not reach statistical significance (adjusted *p* = 0.0561; Figure 7B).

## 3. Discussion

In the present study, we examined the effects of DFAT-EV preparations on inflammatory macrophage responses in vitro and on disease manifestations in a rat model of TMJ-OA in vivo. The established cells exhibited phenotypic characteristics consistent with previously reported DFATs [30,31,33,34]. The isolated preparations exhibited a spherical, membrane-bound vesicular morphology, a mean particle diameter of 177.0 ± 0.6 nm, and the EV-associated markers CD9, CD63, and CD81. These findings were consistent with the basic characteristics of EVs and with previous reports of EVs derived from buccal fat pad-derived DFATs [26,34,35]. However, because markers of cellular or non-vesicular contaminants were not evaluated, the purity of the EV preparations could not be confirmed.

Fluorescence signals associated with the labeled DFAT-EV preparation were observed in RAW264.7 cells; however, because a dye-only control was not included, EV-specific cellular uptake could not be conclusively demonstrated. LPS and IFN-γ stimulation increased NO production and IL-1β, IL-6, and NOS2 expression, whereas DFAT-EV treatment reduced NO production at all tested concentrations and decreased IL-6 mRNA expression and extracellular concentration. IL-1β mRNA expression decreased significantly at 5 and 10 μg/mL but not at 20 μg/mL. At 10 μg/mL, NOS2 expression also decreased without significance. As excessive NO production and increased iNOS, IL-1β, and IL-6 expression have been implicated in joint inflammation and cartilage matrix degradation in TMJ-OA [3,4,6,7,8,10,11,12,37,38,39,40,41], these findings suggest that DFAT-EVs attenuate selected inflammatory macrophage responses. DFAT-EVs also reduced IL-10 concentrations, possibly reflecting attenuation of the compensatory IL-10 response induced by strong inflammatory stimulation [42]. TNF-α increased modestly, whereas IL-4 decreased only at 20 μg/mL, indicating that DFAT-EV effects differed among the inflammatory mediators examined. The TNF-α increase may reflect altered inhibitory feedback associated with lower IL-10 concentrations. Similarly, the IL-4 finding may represent a response specific to the highest DFAT-EV concentration tested. Thus, DFAT-EVs did not uniformly suppress inflammatory responses; the most consistent reductions involved NO production, NOS2 and IL-6 mRNA expression, and IL-6 concentration. Further time courses and mechanistic studies are needed to define the regulation of individual inflammatory mediators.

In vivo, the HWT was significantly higher in the DFAT-EV group than in the OA group at several time points in the 6-week evaluation cohort, whereas no significant difference was detected between these groups in the 4-week evaluation cohort. However, a significant result in one cohort and a non-significant result in another does not by itself demonstrate that the treatment effect varied between the cohorts. DFAT-EV administration attenuated the reduction in mandibular condylar bone volume fraction, although BMD and trabecular microarchitecture did not improve significantly. Similarly, Zhang et al. reported an early increase in BV/TV after intra-articular administration of MSC-derived EVs, whereas broader restoration of osteochondral tissue and subchondral bone architecture became evident after prolonged treatment [27]. Histological evaluation indicated relative preservation of cartilage architecture and matrix in the DFAT-EVs group. The OARSI score was significantly lower in the DFAT-EV group than in the OA group at 4 weeks, whereas the difference did not reach statistical significance at 6 weeks. Furthermore, Kim et al. reported increased ACAN, COL1A1, COL2A1, IGF1, and FGF2 expression after MSC administration, suggesting that MSC-derived paracrine signals may enhance matrix synthesis by resident chondrocytes or progenitor cells [15]. DFAT-EVs may similarly support a matrix-anabolic response in resident chondrocytes and thereby contribute to preservation of the cartilage matrix. However, because cartilage anabolic markers and related signaling pathways were not assessed in the present study, this proposed mechanism remains to be investigated. In addition, intra-articular macrophage activity, inflammatory mediators, and synovial inflammation were not evaluated in vivo. Therefore, the in vitro macrophage findings and the in vivo behavioral and structural findings should be interpreted as parallel observations rather than as evidence of a demonstrated mechanism. Further studies are required to determine whether modulation of inflammatory responses contributes directly to the in vivo effects of DFAT-EVs.

This study has some limitations. The MIA-induced TMJ-OA model may not fully reproduce the chronic and multifactorial pathogenesis of human TMJ-OA, and RAW264.7 cells may not fully reflect the responses of primary intra-articular macrophages. The DFATs were derived from a single donor, which limits the generalizability of the findings and does not account for donor-to-donor variability. DFATs from multiple independent donors should be used in future studies to evaluate donor-to-donor variability and the generalizability of the findings. Cell viability was not evaluated; therefore, changes in cell number or viability may have contributed to the observed reductions in NO and cytokine concentrations. In addition, a dye-only control was not included in the uptake experiment, and a non-EV control processed using the same isolation procedure was not examined. Therefore, the possibility that residual non-vesicular components in the EV preparations contributed to the observed effects cannot be excluded. Markers of cellular or non-vesicular contaminants were not evaluated, and the purity of the EV preparations could therefore not be fully confirmed. Quantitative inter-rater agreement for the histological assessment was also not evaluated. Furthermore, the bioactive cargo of DFAT-EVs was not characterized, and intra-articular macrophage activity, inflammatory mediators, and synovial inflammation were not directly assessed in vivo. Consequently, the in vitro macrophage findings cannot be regarded as a demonstrated mechanism underlying the behavioral and structural findings observed in vivo.

Further work is needed before the therapeutic potential of DFAT-EVs can be established. Studies should be aimed at identifying bioactive molecules in DFAT-EVs, including microRNAs and proteins, and define their target signaling pathways. The effects of DFAT-EVs on synovial inflammation and intra-articular macrophages should also be evaluated directly in vivo. Furthermore, dosing interval and administration timing need to be optimized; long-term joint-protective effects should be examined; and direct comparisons with MSC-derived EVs and existing intra-articular treatments are needed to clarify the therapeutic role of DFAT-EVs.

## 4. Materials and Methods

### 4.1. Cell Culture and Extracellular Vesicle Preparation

#### 4.1.1. Establishment and Culture of DFATs

The human DFATs used in this study were established from buccal fat pad tissue obtained from a single donor during orthognathic surgery at Osaka Dental University Hospital. Before tissue collection, the donor received an explanation of the study and provided written informed consent to participate by signing the informed consent form. This study was approved by the Medical Ethics Committee of Osaka Dental University (Approval No. 111456).

The harvested buccal fat tissue was finely minced and enzymatically digested using 0.1% type I collagenase (FUJIFILM Wako Pure Chemical Corporation, Osaka, Japan) at 37 °C for 60 min. Following digestion, the floating mature adipocyte fraction was collected by centrifugation.

Isolated mature adipocytes were suspended in Dulbecco’s modified Eagle medium (DMEM; FUJIFILM Wako Pure Chemical Corporation) supplemented with 20% fetal bovine serum (FBS) and subjected to ceiling culture in culture flasks completely filled with the medium. The cells were cultured at 37 °C in a humidified atmosphere containing 5% CO_2_. After confirming that the cells had adhered to the inner upper surface of the flask, the flask was inverted, and standard culture was continued. Proliferating fibroblast-like cells were established as DFATs.

Routine expansion culture of DFATs was performed using DMEM supplemented with 15% FBS (Cat. No. 175012; Nichirei Biosciences Inc., Tokyo, Japan) and 1% penicillin-streptomycin (Thermo Fisher Scientific, Waltham, MA, USA) under the same temperature and CO_2_ conditions. Cells at passages 4 to 6 were used for the experiments [30,31,32,33,34].

#### 4.1.2. Flow Cytometric Analysis of DFATs

Surface antigen expression on DFATs was examined using flow cytometry [30,31,34]. Following collection, DFATs were rinsed with phosphate-buffered saline (PBS; FUJIFILM Wako Pure Chemical Corporation), and aliquots containing 1 × 10^5^ cells were prepared for individual analyses. Antibody staining was performed for 30 min at 4 °C under light-protected conditions. The staining panel comprised the following antibodies: anti-CD11b (Cat. No. 301329; BioLegend, San Diego, CA, USA), anti-CD34 (Cat. No. 343503; BioLegend), anti-CD45 (Cat. No. 304005; BioLegend), anti-CD73 (Cat. No. 107942; Invitrogen, Waltham, MA, USA), and anti-CD105 (Cat. No. 323203; BioLegend). A mouse IgG1 κ isotype control antibody (Cat. No. 400109; BioLegend) was used as the isotype control. Flow cytometric measurements were performed with a CytoFLEX flow cytometer, and the resulting data were processed using CytExpert software (version 2.6.0.105) (both from Beckman Coulter Inc., Brea, CA, USA).

#### 4.1.3. Isolation of DFAT-EVs

Each 75 cm^2^ culture flask was inoculated with 1.5 × 10^6^ DFATs. The initial medium was removed 24 h later and replaced with DMEM containing 10% exosome-depleted FBS (Cat. No. FB-1280M/50; Biosera, Cholet, France). Following a further 48–72 h of incubation, the conditioned medium was recovered. A two-step centrifugation procedure was used to clarify the recovered medium: the first centrifugation was performed at 2000× *g* for 30 min, and the second at 10,000× *g* for 30 min. Both steps were conducted at 4 °C to eliminate suspended cells and cellular debris.

EVs were separated from the cleared conditioned medium by size-exclusion chromatography. The sample was first concentrated to 500 μL at 5000× *g* and 4 °C with an Amicon Ultra-15 filter (100-kDa cutoff; Merck Millipore, Burlington, MA, USA). After equilibration of a qEV original/35 nm column (Izon, Christchurch, New Zealand) with PBS, the concentrate was applied and eluted in PBS. An Izon automated fraction collector collected consecutive 500-μL fractions, and fractions 1–4 were pooled as the DFAT-EV preparation used in subsequent experiments [34].

Total protein in the DFAT-EV preparation was quantified with a Micro BCA Protein Assay Kit (Cat. No. 23235; Thermo Fisher Scientific) in accordance with the protocol supplied. The protein-equivalent values obtained from this measurement were used to define the DFAT-EV doses for all subsequent functional assays and animal experiments [34].

#### 4.1.4. Characterization of DFAT-EVs

Particle number and size distribution were measured by nanoparticle tracking using a NanoSight NS300 (Malvern Panalytical, Malvern, UK) and Sample Assistant software (Build 3.4.4). For electron-microscopic examination, the DFAT-EV preparation was subjected to negative contrast staining with 2% uranyl acetate. Images were obtained on an SU9000 microscope (Hitachi High-Tech Corporation, Tokyo, Japan) under an accelerating voltage of 30 kV.

Marker analysis at the single-particle level was conducted on a Flow NanoAnalyzer platform (NanoFCM Inc., Xiamen, China). Fluorescent beads and a silica nanoparticle cocktail were used to calibrate the system according to the supplier’s protocol before data acquisition. Samples were adjusted to a suitable particle concentration by dilution in PBS. Detection of CD9, CD63, and CD81 were performed with fluorescein isothiocyanate-conjugated ExoBrite 490/515 antibodies against CD9 (P003-490), CD63 (P004-490), and CD81 (P005-490), respectively (Biotium, Fremont, CA, USA) [26,34,35].

#### 4.1.5. Fluorescence Labeling and Observation of Labeled DFAT-EV Preparations in RAW264.7 Cells

RAW264.7 murine macrophages obtained from the European Collection of Authenticated Cell Cultures (Salisbury, UK) were used for the fluorescence-labeling experiment.

Membrane labeling of the DFAT-EV preparation was performed with an ExoSparkler Exosome Membrane Labeling Kit-Red (Dojindo Laboratories, Kumamoto, Japan) following the supplier’s protocol. The labeled preparation was applied to RAW264.7 cultures for 4 h. After exposure, the macrophages were fixed with 4% paraformaldehyde, followed by nuclear counterstaining with Hoechst 33342 (Nacalai Tesque, Inc., Kyoto, Japan). The distribution of DFAT-EV-associated fluorescence was visualized using a BZ-X800 fluorescence microscope (Keyence Corporation, Osaka, Japan) [26,34,35,43].

### 4.2. Effects of DFAT-EVs on Inflammatory Responses in RAW264.7 Macrophages

#### 4.2.1. Cell Culture and Inflammatory Stimulation

RAW264.7 macrophages were cultured in DMEM supplemented with 10% FBS (Biosera, Cholet, France) and 1% penicillin-streptomycin (Thermo Fisher Scientific) at 37 °C in a humidified atmosphere containing 5% CO_2_.

To induce inflammation, RAW264.7 macrophages were seeded into 24-well plates at a density of 4 × 10^4^ cells per well. After 24 h, the culture medium was replaced, and the cells were assigned to the following experimental conditions: an unstimulated NC group; a group stimulated with LPS (100 ng/mL; InvivoGen, San Diego, CA, USA) and IFN-γ (20 ng/mL; PeproTech, Rocky Hill, NJ, USA); and DFAT-EV treatment groups stimulated with LPS and IFN-γ and treated with DFAT-EVs at 5, 10, or 20 µg/mL [44,45].

The NO-production and RT-qPCR experiments were independently performed three times on separate occasions, with each experiment using a separately prepared DFAT-EV batch. The ELISA experiments were independently performed four times on separate occasions, with each experiment using a separately prepared DFAT-EV batch. When technical replicate wells were included within an experiment, their values were averaged, and the resulting mean was treated as one independent experimental unit (*n* = 3 for the NO-production and RT-qPCR analyses and *n* = 4 for the ELISA analyses).

#### 4.2.2. NO Assay

Culture supernatants were collected 24 h after inflammatory stimulation. Nitrite concentration was measured as an indirect indicator of NO production [44,45]. Each sample was combined with Griess–Romijn reagent (Lot No. SEN7177; Kishida Chemical Co., Ltd., Osaka, Japan), held for 10 min at 20–25 °C, and read at 540 nm on a SpectraMax M5 plate reader (Molecular Devices, San Jose, CA, USA). Concentrations were interpolated from a sodium-nitrite standard curve (Lot No. C04766P; Kishida Chemical Co., Ltd.).

#### 4.2.3. Quantitative Real-Time PCR (RT-qPCR)

RAW264.7 cells were collected 6 h after inflammatory activation. Total RNA was isolated with the RNeasy Mini Kit (QIAGEN, Venlo, The Netherlands), after which complementary DNA was synthesized using ReverTra Ace qPCR RT Master Mix (TOYOBO Co., Ltd., Osaka, Japan).

Quantitative amplification used THUNDERBIRD Next SYBR qPCR Mix (TOYOBO Co., Ltd.) and a QuantStudio 3 instrument (Thermo Fisher Scientific). FASMAC Co., Ltd. (Atsugi, Kanagawa, Japan) provided primers for NOS2, IL-1β, and IL-6. Expression was referenced to glyceraldehyde-3-phosphate dehydrogenase and calculated relative to the control with the 2^−ΔΔCt^ method [44,45]. The primer sequences used in this study are presented in Table 1.

#### 4.2.4. Enzyme-Linked Immunosorbent Assay (ELISA)

Culture supernatants were obtained 24 h after inflammatory stimulation. The concentrations of IL-6, TNF-α, IL-4, and IL-10 were determined using a Mouse M1/M2 Cytokines Multiplex ELISA Kit (Cat. No. ARG82913; Arigo Biolaboratories, Hsinchu, Taiwan) in accordance with the manufacturer’s instructions. Absorbance at 450 nm was measured using a SpectraMax M5 microplate reader (Molecular Devices, San Jose, CA, USA) [44,45,46].

### 4.3. MIA-Induced Rat Model of TMJ-OA

#### 4.3.1. Experimental Animals and Induction of TMJ-OA

Male Sprague–Dawley rats were 8 weeks old at study entry and were supplied by Shimizu Laboratory Supplies Co., Ltd. (Kyoto, Japan). Before any experimental procedure, the animals underwent an acclimatization period of at least 1 week. During this period and throughout the experiment, they were maintained under controlled temperature and humidity, with a 12-h light/dark schedule and unrestricted access to standard chow and water.

General anesthesia was achieved by intraperitoneal coadministration of midazolam (2 mg/kg; Midazolam Sandoz, Sandoz K.K., Yamagata, Japan), medetomidine hydrochloride (0.15 mg/kg; Domitor, Zenoaq, Fukushima, Japan), and butorphanol tartrate (2.5 mg/kg; Vetorphale, Meiji Seika Pharma Co., Ltd., Tokyo, Japan).

Experimental TMJ-OA was induced by injecting 50 μL of MIA solution (10 mg/mL; 0.5 mg per joint; Code No. 19305-82; Nacalai Tesque, Inc., Kyoto, Japan) into the left temporomandibular joint cavity using a 26-gauge needle.

**Figure 8:**
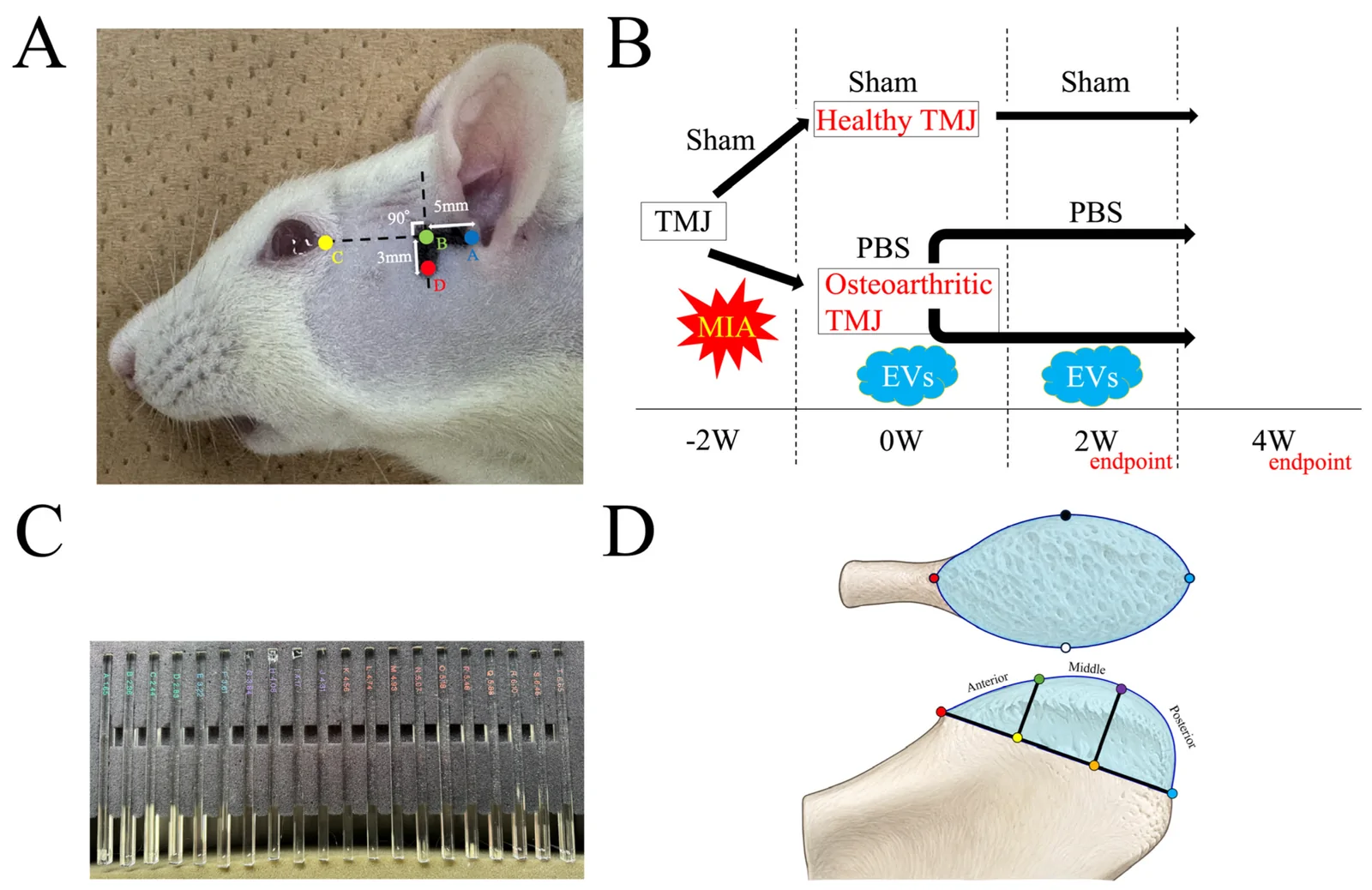
Experimental design of the monosodium iodoacetate (MIA)-induced rat model of temporomandibular joint osteoarthritis (TMJ-OA). (**A**) Anatomical landmarks and injection site for intra-articular administration into the left TMJ. The needle was inserted at a 45° angle in the superior, anterior, and medial directions. (**B**) Experimental groups and treatment schedule. TMJ-OA was induced by intra-articular administration of MIA (0.5 mg/50 μL). Beginning 2 weeks after MIA administration (Week 0), phosphate-buffered saline or DFAT-EVs were administered intra-articularly at 2-week intervals. The 4-week evaluation cohort received one injection, whereas the 6-week evaluation cohort received two injections. (**C**) Semmes–Weinstein monofilaments used to measure the head withdrawal threshold. (**D**) Schematic representation of the evaluation regions of the mandibular condyle used for histological and micro-computed tomography analyses. The upper and lower images show the axial and sagittal views, respectively. In the sagittal view, the mandibular condyle was divided into anterior, middle, and posterior regions. The blue-shaded regions indicate the mandibular condylar volume of interest (VOI) used for quantitative micro-CT analysis. Quantitative assessment was performed on the trabecular bone compartment within this VOI, excluding cortical bone and mineralized cartilage.

Joint access was determined from external anatomical landmarks. Points A and C corresponded to the preauricular region and posterior corner of the orbit, respectively, and a reference line was established between them. Point B was placed 5 mm anterior to point A. The needle-entry position, designated point D, was set 3 mm inferior to point B along a perpendicular line. The needle was advanced from point D toward the superior, anterior, and medial aspects at an angle of approximately 45°, after which the MIA solution was delivered into the joint cavity [47] (Figure 8A).

The experimental protocol was authorized by the Animal Care and Use Committee of Osaka Dental University (Approval No. 26-03004), and all procedures were performed in compliance with the applicable guidelines.

#### 4.3.2. Intra-Articular Administration of DFAT-EVs

Separate cohorts were established for evaluation 4 and 6 weeks after MIA administration. Within each cohort, animals were assigned to the Sham, OA, and DFAT-EVs groups without deliberate selection; however, no formal method was used to generate a randomization sequence. Each group comprised five animals at each endpoint. Rats assigned to the OA and DFAT-EV groups received MIA in the left temporomandibular joint cavity, whereas rats in the Sham group underwent needle insertion at the same site without MIA injection. No formal a priori sample-size calculation was performed. No animals were excluded during the experiment, and all animals were included in the final analysis.

At 2 weeks after MIA administration, the DFAT-EV group received DFAT-EVs (100 μg/mL, 50 μL), whereas the OA group received PBS (50 μL); both preparations were injected into the left temporomandibular joint cavity. The day of the first DFAT-EV or PBS injection, corresponding to 2 weeks after MIA administration, was designated as Week 0 on the treatment timeline. DFAT-EVs or PBS were administered at 2-week intervals beginning 2 weeks after MIA administration. The 4-week evaluation cohort received one injection at 2 weeks, whereas the 6-week cohort received two injections at 2 and 4 weeks after MIA administration [48] (Figure 8B).

#### 4.3.3. HWT

HWT was assessed once per week, beginning on the day of MIA administration. Measurements were continued through 4 weeks after MIA administration in the 4-week evaluation cohort and through 6 weeks after MIA administration in the 6-week evaluation cohort.

Responses to calibrated Semmes–Weinstein monofilaments (KN-935-A; Natsume Seisakusho Co., Ltd., Tokyo, Japan) were used to quantify mechanical hypersensitivity [36] (Figure 8C). The filaments were applied perpendicular to the preauricular skin over the left temporomandibular joint in order of increasing force. Each filament was applied three times, and the lowest force that produced a head withdrawal response in all three applications was recorded as the HWT. The investigators conducting the HWT measurements were not blinded to group allocation.

#### 4.3.4. Micro-CT Analysis

At each evaluation time point, five specimens from each group were examined by micro-CT using a SKYSCAN 1275 system (Bruker, Billerica, MA, USA). Each specimen was secured in a 25-mm-diameter holder and scanned at a tube voltage of 65 kV and tube current of 85 μA using a 1-mm aluminum filter. The reconstructed voxel size was set at 20 μm for all specimens. The acquired projection images were reconstructed using NRecon software (version 1.7.4.2; Bruker), and the reconstructed images were reoriented in the coronal plane using DataViewer software (version 1.5.6.2; Bruker).

The volume of interest (VOI) encompassed the mandibular condyle from its medial to lateral margins. On each section, the region extending from the anterior margin to the posterior margin of the mandibular condyle was defined as the region of interest. For regional identification, the mandibular condyle was divided into anterior, middle, and posterior regions [36,48,49] (Figure 8D).

Quantitative measurements were obtained using CTAn software (version 1.17.4.2; Bruker). Within the defined mandibular condylar VOI, the trabecular bone compartment was used for quantitative analysis, with cortical bone and mineralized cartilage excluded. Segmentation was performed using the automatic thresholding function in CTAn. The bone microstructural parameters assessed within the defined mandibular condylar VOI were Tb.N, Tb.Th, Tb.Sp, BV/TV, and BMD [36,48,49,50]. Three-dimensional images were generated using CTvox software (version 3.3.0r1403; Bruker). The investigators conducting the micro-CT analyses were not blinded to group allocation.

### 4.4. Histological Evaluation

#### Preparation and Evaluation of Histological Specimens

At the 4- and 6-week endpoints, rats were euthanized by an overdose of inhaled isoflurane (Cat. No. 099-06571; FUJIFILM Wako Pure Chemical Corporation). The left temporomandibular joints were then excised and immersed in 10% neutral-buffered formalin. Decalcification was performed by incubating the fixed specimens in 10% ethylenediaminetetraacetic acid at room temperature for 10 d. After decalcification, the samples were processed for paraffin embedding. Serial sagittal sections were prepared from the embedded specimens at a thickness of 2.5 μm.

Sections were stained with hematoxylin and eosin or Safranin O/Fast Green. Images of the anterior condylar region were acquired using a BZ-X800 microscope (Keyence Corporation). Cartilage degeneration was graded according to the OARSI scoring system [51].

For each animal, one sagittal section was selected at a comparable anatomical level based on the anatomical position and morphology of the mandibular condyle. Histological section selection and OARSI assessment were independently performed by three evaluators who were blinded to the experimental groups. The OARSI grade ranged from 0 to 6, with grade 0 representing intact articular cartilage with normal morphology. The stage ranged from 0 to 4, with stage 0 indicating no OA involvement. The OARSI score was calculated by multiplying the grade representing the severity of cartilage degeneration by the stage representing the extent of the lesion, as follows: OARSI score = Grade × Stage. The median of the three evaluators’ scores was used as the final OARSI score for each animal. Quantitative inter-rater agreement was not assessed.

### 4.5. Statistical Analysis

Results are reported as the mean ± standard deviation, except for OARSI scores, which are presented as the median and interquartile range. GraphPad Prism 10.1.2 (GraphPad Software, San Diego, CA, USA) was used for all analyses. For the in vitro analyses, *n* represents the number of independent experiments performed on separate occasions (*n* = 3 for NO production and gene expression analyses and *n* = 4 for cytokine measurements). Differences among groups in NO production, gene expression, cytokine concentrations, and micro-CT parameters were analyzed using one-way analysis of variance (ANOVA), followed by Sidak’s multiple-comparisons test. OARSI scores were analyzed using the Kruskal–Wallis test, followed by Dunn’s multiple-comparisons test. Longitudinal changes in HWT were analyzed using two-way repeated-measures ANOVA, with treatment group as the between-subject factor and time as the within-subject repeated factor, followed by Tukey’s multiple-comparisons test. The individual animal was considered the experimental unit for the longitudinal HWT analysis. A *p*-value < 0.05 was considered statistically significant.

## 5. Conclusions

In this exploratory study, treatment with DFAT-EV preparations was associated with changes in selected components of inflammatory macrophage responses in vitro and with improvements in selected behavioral and structural outcomes in experimental TMJ-OA. Because macrophage activity and synovial inflammation were not directly assessed in vivo, the in vitro and in vivo findings should be regarded as parallel observations rather than as evidence of a demonstrated macrophage-mediated mechanism. In addition, the single-donor design, absence of cell-viability assessment, and lack of appropriate EV-specific controls limit the interpretation of these findings and preclude definitive attribution of the observed effects to EV-specific activity. These findings support further evaluation of DFAT-EV preparations in experimental TMJ-OA.

## Figures and Tables

**Table 1:** Primer sequences used for quantitative real-time PCR.

Gene	Forward Primer Sequence (5′–3′)	Reverse Primer Sequence (5′–3′)
NOS2	GAGACAGGGAAGTCTGAAGCAC	CCAGCAGTAGTTGCTCCTCTTC
IL-1β	TGGACCTTCCAGGATGAGGACA	GTTCATCTCGGAGCCTGTAGTG
IL-6	TACCACTTCACAAGTCGGAGGC	CTGCAAGTGCATCATCGTTGTTC
GAPDH	CATCACTGCCACCCAGAAGACTG	ATGCCAGTGAGCTTCCCGTTCAG

GAPDH, glyceraldehyde-3-phosphate dehydrogenase.

## Data Availability

The data supporting the findings of this study are available from the corresponding author, Y.N., upon reasonable request.
